# Lack of knowledge is the leading key for the growing cervical cancer incidents in Bangladesh: A population based, cross-sectional study

**DOI:** 10.1371/journal.pgph.0000149

**Published:** 2022-01-04

**Authors:** Nur E. Alam, Md. Shariful Islam, Fabia Rayyan, Humaira Nur Ifa, Md Imam Ul Khabir, Kamal Chowdhury, A. K. M. Mohiuddin

**Affiliations:** 1 Department of Biotechnology and Genetic Engineering, Mawlana Bhashani Science and Technology University, Tangail, Bangladesh; 2 Department of Biology, University of Kentucky, Lexington, KY, United States of America; 3 Department of Biological Science, Alabama State University, Montgomery, AL, United States of America; 4 Department of Biology, Claflin University, Orangeburg, SC, United States of America; University of Nebraska Medical Center Pharmacy Practice: University of Nebraska Medical Center College of Pharmacy, UNITED STATES

## Abstract

**Introduction:**

Cervical cancer is the second most common gynecological cancer in Bangladesh. Lack of awareness of screening methods, risk factors, and symptoms may lead to presenting most cervical cancers at an advanced stage. We investigated knowledge and awareness of cervical cancer (CCa) among females at the Sheikh Hasina Medical College (SHMC) of Tangail district in Bangladesh.

**Methods:**

A cross sectional survey was conducted to collect data via a structured questionnaire from SHMC during the period of February 2019 to January 2020. Data on socio-demographic characteristics and knowledge of cervical cancer were collected. Multivariable logistic regression models were used to identify factors associated with having heard and knowledge of cervical cancer. A *p*-value <0.05 was considered significant.

**Result:**

Of all the interviews conducted, only 45.2% (493/1090) had heard of cervical cancer as a disease. Women were more likely to be aware of CCa if they were lived in urban areas, had higher education (university level education) and belong to high income families. The study revealed evidence of significant association between marital, literacy, residence and socio-economic status with women’s knowledge on cervical cancer (*p*< 0.05).

**Conclusion:**

This study serves to highlight that there was impoverished knowledge about cervical cancer among Bangladeshi women. Hence, this indicates the government should take proper steps to raise awareness and knowledge levels via educational programs and health counseling.

## Introduction

Cancer of the cervix is considered as a major public health problem of women across the globe. Globally, it is the fourth most common malignancy- in terms of both incidence and mortality; among women. In 2020, an estimated 604,127 cases and 341,831 deaths were reported due to cervical cancer (CCa) worldwide [1]. According to World Health Organization (WHO), if additional action will not be taken, the annual number of new cases is expected to be raised to 700,000 and deaths are projected to increase to 400,000 between 2018 and 2030 [2].

The South Asian region is home to one fourth of the incidence of cervical cancer, where more than 130,000 new cases are reported every year [3, 4]. In Bangladesh, there is no national central cancer registry that can provide the complete nationwide data [5]. Therefore, the actual scenario of cervical cancer is largely unknown. However, according to Global Cancer Incidence, Mortality and Prevalence (GLOBOCAN), CCa is the second most common gynecological cancer with an estimated age‐standardized incidence and mortality rates of the disease were approximately 10.6 and 6.7 per 100,000 women, respectively, in 2020. Currently, an estimated 8,268 women are diagnosed and 4,971 women die of CCa every year in Bangladesh [6]. Like other less developed countries, the higher rate of mortality due to cervical cancer in Bangladesh has been attributed to several factors such as lack of awareness about its symptoms, risk factors, screening programs, and preventive measures [7]. A study revealed that less than 10% of participants had in-depth knowledge of the causes and possible preventive measures of cervical cancer [8].

The most important risk factor associated with cervical cancer is the infection of Human Papillomavirus (HPV) which is transmitted through sexual contact [9]. It is estimated that infection with HPV is associated with 70% of cervical cancer cases [10]. In addition to HPV, other known risk factors include marriage at an early age, multiple sexual partners, prolonged use of oral contraceptive pills, multiple childbirths, immunosuppressants and specific dietary factors [10, 11].

Unlike other cancers, the morbidity and mortality of CCa could be reduced through early detection and treatment. Regular screening programs like Pap smear, HPV DNA test, and visual inspection of the cervix with acetic acid (VIA) reduced both the incidence and mortality of cervical cancer in the developed countries [12]. In the United States, the death rate from cervical cancer has reduced to 50% over the last four decades by an increased rate of using Pap test [9]. But in Bangladesh, cervical cancer is being detected at a much later stage because it is too late when women come for diagnosis. An earlier study found that despite Bangladeshi women admitted Pap testing could reduce their risk of cervical cancer, most women neglected screening due to inadequate awareness and lack of facilities [7].

Therefore, the present study aims to assess the knowledge and awareness related to cervical cancer among females in Tangail district of Bangladesh.

## Materials and methods

### Participants and study area

This is a cross-sectional descriptive study conducted to investigate participants’ knowledge about risk factors associated with cervical cancer from February 2019 to January 2020 on 1,090 women in Sheikh Hasina Medical College, Tangail, Bangladesh.

Bengali adult females age 15–75 years attending the Department of Gynaecology and Obstetrics, Sheikh Hasina Medical College, Tangail, Bangladesh for routine services or accompanying patients visited the hospital were included in the study by a nonprobability convenience sampling. Non-Bengali females and women age less than 15 years or more than 75 years were excluded.

The respondents were given an explanation of the objectives and benefits of the study. Participants provided verbal consent during the clinical visits and face to face clinical interview were conducted accordingly. Extra verbal consent was also obtained from guardians for respondents in case of under the legal age. Those who were not willing to participate, were not given the questionnaires. The data collection was anonymous. This study was conducted according to the WHO and Bangladesh Medical Research Council (BMRC) guidelines of ethical consideration. Respondent’s right to refuse and withdraw from study any time was accepted. Confidentiality of the respondents was strictly maintained.

### Sample size

We utilized the following formula to calculate the sample size:

$$n=\frac{Z^{2}\;P(1–P)}{d^{2}}$$

Here n = sample size, Z = Z statistic for a level of confidence, P = expected prevalence or proportion and d = precision [13]. An assumption of 50% prevalence of good knowledge, 3% allowable error, and 95% confidence intervals were taken for calculating the minimal sample size which was found 1,068. The sample size was increased by 2% to allow for non-sampling error, particularly nonresponse error. Thus, the final estimated sample size was 1,090 which was considered adequate to fulfill the objectives of our study.

### Questionnaire content

The interview was conducted via a self-administrative close-ended questionnaire. The questionnaire was first developed in English and also translated into Bengali according to Bangladesh Medical Research Council (BMRC) guidelines of ethical consideration. The questionnaire was comprised of 15 questions and was divided into five sections. The first section consisted of socio demographic variables which included marital status, age, weight, education, living place, religion, socio-economic level. The second and third sections assessed the knowledge of the participant regarding risk factors and symptoms of cervical cancer, respectively. The fourth and fifth sections consisted of participant’s knowledge on screening and treatments, respectively.

A knowledge score was calculated by summing the responses for each participant who reported having heard of cervical cancer. They were given two points for any two correct responses for symptoms, risk factor, treatment and screening associated with cervical cancer. The total score ranged between 0 and 8. According to our criteria, a total score of 0 to 4 was categorized as insufficient knowledge and scores greater than or equal to 5 were considered sufficient knowledge.

Cronbach’s Alpha was used to assess the reliability coefficient which is a measure of the internal consistency of the questionnaire. The Cronbach’s alpha coefficient was 0.807 for the questionnaire where the value >0.7 is considered acceptable [14].

### Statistical analysis

All statistical analyses were performed using Statistical packages for social sciences (SPSS) version 20 statistical software. Categorical variables were described using frequencies and percentages, and continuous variables were summarized using means and standard deviations. Socio-demographic characteristics (e.g. living place, gender, literacy status) had been considered as independent variables while ever heard of cervical cancer or Knowledge (e.g. risk factors, screenings, symptoms) as outcome variables. Multivariable logistic regression models were generated to assess factors associated with knowledge of cervical cancer. Adjusted odds ratios (aORs) and its 95% confidence intervals (CIs) were estimated. First, variables of interest were assessed using univariate analysis. Any factor that provided a univariate *p*-value ≤ 0.05 was entered into the multivariable analysis. The following variables were adjusted for in the models: marital status, living place, literacy, religion and socio-economic status. Collinearity was assessed using the variance inflation factor (VIF) to ensure a strong linear relationship among independent variables included in the model was not present. The goodness of fit of the model was checked using the Hosmer Lemeshow (H-L) test. *P*-values of *<* 0.05 were considered significant.

### Ethical consideration

Before initiating the study, approval was obtained from the Department of Biotechnology and Genetic Engineering, Mawlana Bhashani Science and Technology University, Tangail-1902, Bangladesh (Ref: MBSTU/BGE/Research project (87)/2009(105)).

## Results

### Socio-demographic characteristics

A total of 1,240 eligible women were approached and 1,090 were interviewed completely, giving a response rate of 87.9%. The ages of the women were between 15 to 75 years. The mean age of the participants was 33.09 ± SD 12.32 year. Most of them were married and resided in rural areas. The majority (88.6%) were Muslims and 56.8% belonged to middle income families (**Table 1**).

**Table 1:** Socio-demographic characteristics of female respondents (n = 1,090).

Variables	n (%)
**Marital status**	
Married	848 (77.8)
Unmarried	242 (22.2)
**Age**	
15–25	344 (31.6)
26–35	356 (32.7)
36–45	217 (19.9)
46–55	109 (10)
56–65	47 (4.3)
66–75	17 (1.6)
Mean ± SD	33.09 ± 12.32
**Living place**	
Rural	941 (86.3)
Urban	149 (13.7)
**Literacy status**	
Undergraduate	227 (20.8)
Graduate	88 (8.1)
Others*	775 (71.1)
**Religion**	
Muslim	889 (88.3)
Hindu	108 (10.7)
Others	10 (1)
**Socio economic status**	
Low income (<15000 BDT/Month)	456 (41.8)
Middle income (15000 –<1,00,000 BDT/Month)	619 (56.8)
High income (>1,00,000 BDT/Month)	15 (1.4)

Literacy status: Others

* include primary/secondary/ no formal education.

### Awareness about cervical cancer

About 493 females (45.2%) were aware of the terminology “cervical cancer” as a disease (**Table 2**). The participants (597/1090) who did not know cervical cancer as a disease were excluded from further questioning.

**Table 2:** Univariate and multivariable analyses of factors associated with ever heard of cervical cancer (Q8).

Variables	Ever heard of cervical cancer		Univariate			Multivariate	
	Yes (n = 493), n (%)	No (n = 597), n (%)	OR (95% CI)	*p* value	OR (95% CI)		*p* value
**Marital status**							
Married	367 (43.3)	481 (56.7)	Ref.	**0.016**	Ref.		0.895
Unmarried	126 (52.1)	116 (47.9)	0.702 (0.527–0.935)		1.022 (0.739–1.415)		
**Living place**							
Rural	388 (41.2)	553 (58.8)	Ref.	**<0.001**	Ref.		**<0.001**
Urban	105 (70.5)	44 (29.5)	0.294 (0.202–0.428)		0.412 (0.276–0.614)		
**Literacy status**							
Undergraduate	136 (59.9)	91 (40.1)	Ref.	**<0.001**	Ref.		**<0.001**
Graduate	52 (59.1)	36 (40.9)	2.303 (1.703–3.115)		2.076 (1.475–2.923)		
Others	305 (39.4)	443 (60.6)	2.226 (1.421–3.486)		1.627 (1.002–2.641)		
**Religion**							
Muslim	450 (46.6)	516 (53.4)	Ref.	**0.028**	Ref.		**0.016**
Hindu	38 (33.3)	76 (66.7)	0.872 (0.251–3.032)		0.664 (0.186–2.367)		
Others	5 (50)	5 (50)	0.5 (0.136–1.833)		0.36 (0.095–1.367)		
**Socio economic status**							
Low income	163 (35.7)	293 (64.3)	Ref.	**<0.001**	Ref.		**0.024**
Middle income	320 (51.7)	299 (48.3)	0.278 (0.093–0.828)		0.524 (0.164–1.674)		
High income	10 (66.7)	5 (33.3)	0.535 (0.181–1.584)		0.753 (0.239–2.370)		

Bold shows factors that were significant.

Women were more likely to be aware of CCa if they were lived in urban areas (adjusted odds ratio [aOR]: 0.412; 95% confidence interval [CI]: 0.276–0.614) than those who were lived in rural areas in Bangladesh. Women who had higher education (university level education) (aOR: 2.076; 95% CI: 1.475–2.923) were more than 1.5 times more informed than those who had primary/ secondary education, or did not have any formal schooling. All of the socio-demographic variables except marital status showed significant relationship with having heard of cervical cancer (*p* <0.05). The H-L p value for the model was 0.261.

### Knowledge of cervical cancer symptoms and risk factors

Knowledge about cervical cancer symptoms and risk factors in women are assessed in both rural and urban areas. Majority of the respondents were aware of “bleeding in between periods” (identified by 54.4% and 53.3% of rural and urban individuals, respectively), “menstrual period that are longer than as usual” (identified by 50.5% and 44.8% of rural and urban individuals, respectively) and “bleeding after menopause” (identified by 33.2% and 42.9% of rural and urban individuals, respectively).

In this survey, we found that women who resided in urban areas were more aware of the risk factors than the respondents resided in rural areas. In the rural area, the most commonly known risk factors were long term use of contraceptive pill (44.6%), starting to have sex at a young age (before age 17) (38.7%), having many children (36.9%) and having many sexual partners (36.1%). The most known risk factors in urban individuals were starting to have sex at a young age (before age 17) (57.1%), having many sexual partners (52.4%) and having many children (48.6%). Not going for a regular Pap smear test and infection with HPV were the least known risk factors in both rural and urban areas (**Table 3**).

**Table 3:** Knowledge about symptoms and risk factors of cervical cancer.

Symptoms	Rural	%	Urban	%	OR (95% CI)
Bleeding in between periods	211	54.4	56	53.3	1.043 (0.677–1.607)
Blood in the stool or urine	115	29.6	28	26.7	1.158 (0.714–1.88)
Menstrual period that are longer than as usual	196	50.5	47	44.8	1.26 (0.817–1.943)
Persistent lower back pain & diarrhea	67	17.3	16	15.2	1.161 (0.641–2.102)
Bleeding after menopause	129	33.2	45	42.9	0.664 (0.428–1.032)
Swollen legs, bone fractures	54	13.9	12	11.4	1.253 (0.643–2.44)
Persistent vaginal discharge that smells unpleasant	108	27.8	38	36.2	0.68 (0.431–1.073)
**Risk factors**					
Weakened immune system	46	11.9	33	31.4	0.293 (0.175–0.491)
Infection with HPV	60	15.5	34	32.4	0.382 (0.233–0.625)
Long term use of contraceptive pill	173	44.6	48	45.7	0.956 (0.62–1.473)
Having many children	143	36.9	51	48.6	0.618 (0.4–0.955)
Having many sexual partners	140	36.1	55	52.4	0.513 (0.332–0.793)
Not going for regular Pap smear test	25	6.4	17	16.2	0.357 (0.185–0.689)
Starting to have sex at a young age (before age 17)	150	38.7	60	57.1	0.473 (0.305 - .732)

### Knowledge about screening and treatment

Half of the participants (246/493) were aware about the screening test for cervical cancer and out of these eighteen (3.7%) respondents said that it could be detected by Colposcopy and 8.7% of women knew about Pap smear test. About 38.9% and 31.6% of women knew about ultrasound and MRI screening, respectively as those procedures are normally used in multiple disease diagnosis processes (**Table 4**).

**Table 4:** Participants knowledge about the screening methods of cervical cancer.

Screening methods	Yes	No
	n = (246) (%)	n = (247) (%)
Colposcopy	18 (3.7)	475 (96.3)
Ultrasound	192 (38.9)	301 (61.1)
Pelvic Exam	25 (5.1)	468 (94.9)
Pap smear	43 (8.7)	450 (91.3)
HPV (Human Papillomavirus) Test	42 (8.5)	451 (91.5)
Endocervical curettage	23 (4.7)	470 (95.3)
Cone Biopsy	40 (8.1)	453 (91.9)
CT scan	164 (33.3)	329 (66.7)
MRI	156 (31.6)	337 (68.4)

**Fig 1:**
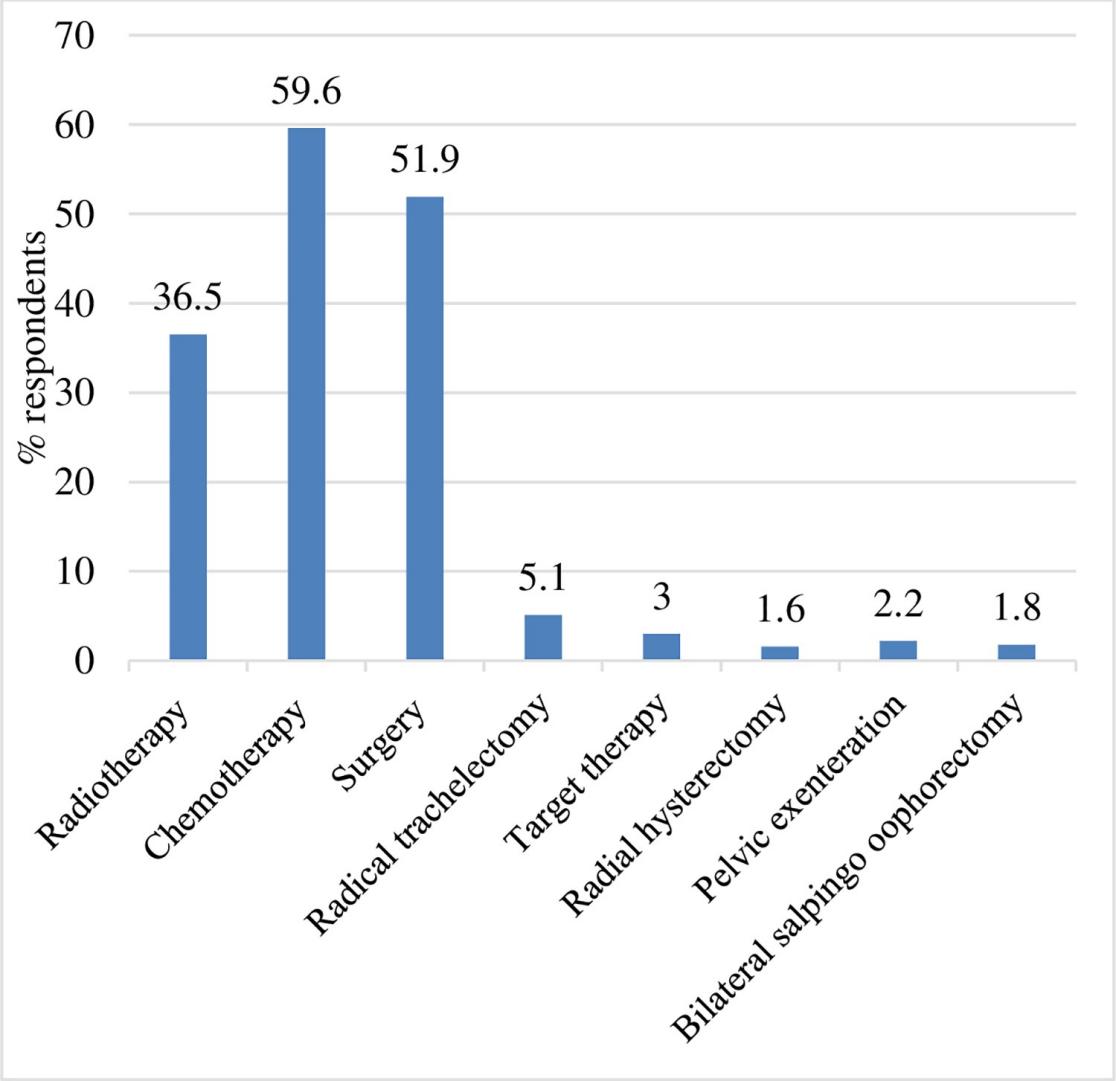
Participants knowledge about cervical cancer treatment.

Participants were also asked about the knowledge of cervical cancer treatment. Fifty nine percent had the opinion that chemotherapy is required to cure cervical cancer, whereas 51.9% were in favor of surgery. The other treatments included Bilateral salpingo oophorectomy (1.8%), Pelvic exenteration (2.2%) and Radical trachelectomy (5.1%) (**Fig 1**).

### Factors associated with cervical cancer knowledge

**Table 5** represents the associations between the socio-demographic variables and knowledge on cervical cancer. The respondents who were unmarried (adjusted odds ratio [aOR]: 0.609; 95% confidence interval [CI]: 0.426–0.871) and lived in urban areas (aOR: 0.461; 95% CI: 0.31–0.686) were more likely to have cervical cancer knowledge than those who were married and lived in rural areas. Respondents with a qualification of graduate (aOR: 1.315; 95% CI: 0.894–1.934) were more knowledgeable than those who had primary education or did not have formal schooling. Moreover, middle economic respondents (aOR: 1.448; 95% CI: 0.361–5.808) were 2 times more informed than lower economic respondents. However, women’s knowledge score on cervical cancer varied significantly by marital status (*p* = 0.007), the area of residence (*p*<0.001), level of education (*p* = 0.025), and socio-economic status (*p* = 0.005) (**Table 5**). The H-L p value for the model was 0.173.

**Table 5:** Univariate and multivariable analyses of factors associated with knowledge on cervical cancer.

Variables	Knowledge on cervical cancer		Univariate		Multivariate	
	Insufficient n (%)	Sufficient n (%)	OR (95% CI)	*p* value	OR (95% CI)	*p* value
**Marital status**						
Married	697 (82.2)	151 (17.8)	Ref.	**<0.001**	Ref.	**0.007**
Unmarried	164 (67.8)	78 (32.2)	0.456 (0.33–0.628)		0.609 (0.426–0.871)	
**Living place**						
Rural	771 (81.9)	170 (18.1)	Ref.	**<0.001**	Ref.	**<0.001**
Urban	90 (60.4)	59 (39.6)	0.336 (0.233–0.486)		0.461 (0.31–0.686)	
**Literacy status**						
Undergraduate	161 (70.9)	66 (29.1)	Ref.	**<0.001**	Ref.	**0.025**
Graduate	55 (62.5)	33 (37.5)	2.034 (1.444–2.866)		1.315 (0.894–1.934)	
Others	645 (83.2)	130 (16.8)	2.977 (1.859–4.767)		1.977 (1.187–3.291)	
**Religion**						
Muslim	753 (78)	213 (22)	Ref.	0.067	Ref.	-
Hindu	99 (86.8)	15 (13.2)	2.546 (0.321–20.207)		-	
Others	9 (90)	1 (10)	1.364 (0.161–11.546)		-	
**Socio economic status**						
Low income	396 (86.8)	60 (13.2)	Ref.	**<0.001**	Ref.	**0.005**
Middle income	453 (73.2)	166 (26.8)	0.606 (0.166–2.21)		1.448 (0.361–5.808)	
High income	12 (80)	3 (20)	1.466 (0.409–5.259)		2.478 (0.64–9.602)	

## Discussion

Most of the women in this study were unable to recognize cervical cancer as a major public health problem. A total of 45.2% who participated in the survey reported they were aware of the term “cervical cancer.” This study findings were similar to other studies conducted in India, Pakistan and Zambia where 53.2, 51.3 and 36.8% of participants respectively, were knowledgeable of the term cervical cancer [12, 15, 16]. A study conducted among middle aged women in Bangladesh reported that 81% of women knew about cervical cancer [17]. The association between literacy and living area with knowledge of CCa is not surprising at all.

Long-term use of oral contraceptives, early onset of sexual activity and multiple sexual partners were most recognized risk factors by women in this study. The findings were consistent with other studies carried out in India, Bhutan, Cameroon and Uganda [12, 18–20]. However, an alarming finding of our study was that awareness of HPV as a risk factor was very low in our study population in both rural (15.5%) and urban (32.4%) areas, even though 98% of cervical cancer in Indian region is due to HPV infection [21]. A previous study in Hong Kong mentioned that 90% of the participants had never heard of HPV [22]. In contrast to ours, some previous studies reported being infected with the HPV as the most widely known risk factor [20, 23, 24]. Regarding awareness of cervical cancer symptoms, the most recognized symptoms in our sample population were “bleeding in between periods,” “menstrual periods that are longer than as usual” and “bleeding after menopause” (54.2, 49.3 and 35.3% respectively). Previous study conducted in Pakistan and Ghana where 56.8 and 56.1% respondents, respectively recognized “bleeding in-between period” as a symptom of CCa [15, 23]. Our results suggest that in order to diminish CCa morbidity and mortality, more efforts are needed to educate the general public, especially women regarding CCa symptoms.

Cervical cancer is readily preventable through the widespread introduction of cytological screening programs. In this study, only 49.9% were aware of the screening program and most of them knew about ultrasound and MRI screening. Merely 8.7% of women knew that Pap smear is a screening test for cervical cancer. Similar finding was reported in a previous study in India where 7% reported that CCa could be detected by Pap smear [25]. A recent research study carried out in Pakistan showed that only 34.2% were aware of Pap smear test [15]. A study conducted in Chittagong Medical College Hospital, Bangladesh for evaluating knowledge about VIA test and only 22.44% of the participants identified it as a screening test for cervical cancer [26]. The study result depicts a lack of knowledge regarding CCa screening which may explain why most of the cervical cancers in Bangladesh remain diagnosed at an advanced stage.

The findings from the present study showed that a significant relationship existed between knowledge of participants and their socioeconomic status. In this study, unmarried women had better knowledge as compared to married women. Participant’s knowledge regarding CCa was also found to be significantly associated with education; women with a university-level education had better knowledge scores than the women from other education levels. This is analogous with the findings of two previous studies conducted in Pakistan and the Maldives in which women’s knowledge improved with higher education [27, 28]. However, our study indicates that the less educated women have a more prominent absence of awareness regarding CCa.

Cervical cancer is a rising concern for Bangladesh. To get rid of the burden of cervical cancer, there is a need to make aware the general people and organize routine screening tests all over Bangladesh. More than 2.4 million female workers are employed in 4825 garment factories in Bangladesh. If the government and the garment industry authorities could jointly provide cytological screening like VIA testing in all garment factories, it would be a huge step towards widespread cervical cancer screening in Bangladesh [29].

The healthcare system of cervical cancer is very low in Bangladesh. The main reason behind the poor access to healthcare and diagnosis center is the financial crisis of mass population. There is no nationwide cervical cancer registry report to track the pre-diagnosis to post-diagnosis report of cervical cancer patients in Bangladesh. So, it’s an urgent issue to make guidelines for the cervical cancer awareness, screening and proper way of treatment including the healthcare system up gradation.

## Strength and limitation

Our study recommends an urgent need to educate women in this locality on different aspects of cervical cancer. Special consideration should be applied to rural, illiterate, lower-income women for the enhancement of knowledge about this lethal cancer.

However, selection of respondents in this study was based on convenience sampling, this might have caused some bias in the representation of our respondents. As the survey questionnaire is based on multiple choice, it is possible that some respondents might provide socially desirable responses to some questions.

## Conclusion

The majority of the women in our study never heard about cervical cancer. The rest of them have poor knowledge about cervical cancer. This unfamiliarity and lack of knowledge about cervical cancer could be a very great threat and will make the situation worse. So, spreading the knowledge is a must for achieving the goal of reducing the mortality rates associated with cervical cancer. A news of hope that most of the females in our study who have heard or never heard about cervical cancer have willingly participated in this survey which indicates that they are really eager to know and gather knowledge about this cancer.

## Supporting information

S1 Questionnaire Knowledge towards cervical cancer survey questionnaire. (DOCX)

S1 Data Survey data files SPSS. (SAV)
