# Supplementary material for: Lack of knowledge is the leading key for the growing cervical cancer incidents in Bangladesh: A population based, cross-sectional study
Source: PLOS Glob Public Health. 2022 Jan 4;2(1):e0000149. doi: 10.1371/journal.pgph.0000149 (PMC10021366; doi:10.1371/journal.pgph.0000149)
Supplement: S1 Questionnaire — (DOCX) [file pgph.0000149.s001.docx]

**Sample ID: Form #**

***Socio‐demographic profile (আর্থ-সামাজিক অবস্থা)***

1. Marital Status (বৈবাহিক অবস্থা**)**

| o Married (বিবাহিত) | o Unmarried (অবিবাহিত) |
| --- | --- |

2. Age (year) **(বয়স)**

| o 15-25 (১৫-২৫) | o 26-35 (২৬-৩৫) | o 36-45 (৩৬ - ৪৫) |
| --- | --- | --- |
| o 46-55 (৪৬-৫৫) | o 56-65 (৫৬-৬৫) | o 66-75 (৬৬ - ৭৫) |

3. Weight (Kg) **(ওজন)**

| o 35‐50 (৩৫-৫০) | o 51‐60 (৫১-৬০) | o 61‐70 (৬১-৭০) |
| --- | --- | --- |

4. Living place (বাসস্থান)

| o Rural (গ্রাম) | o Urban (শহর) |
| --- | --- |

**5.** Study level (শিক্ষাগত যোগ্যতা)

| o Undergraduate (স্নাতকার্থী) | o Graduate (স্নাতক) | o Others (অন্যান্য) |
| --- | --- | --- |

**6.** Religion (ধর্ম)

| o Muslim (মুসলিম) | o Hindu (হিন্দু) | o Others (অন্যান্য) |
| --- | --- | --- |

7. Socio‐economic level **(**আর্থ-সামাজিক অবস্থান**)**

| o Low (নিম্ন) | o Middle (মধ্যম) | o High (উচ্চ) |
| --- | --- | --- |

8. Do you ever heard about cervical cancer (আপনি কি জরায়ু ক্যান্সার সম্পর্কে শুনেছেন?)

| - Yes (হ্যাঁ) | - No (না) |
| --- | --- |

***Knowledge about the Risk factors of Cervical Cancer*** (***জরায়ু ক্যান্সারের ঝুঁকিপূর্ণ কারণ সম্পর্কে জ্ঞান)***

9. Do you have any knowledge about the risk factors of cervical cancer? (সার্ভিকাল ক্যান্সারের ঝুঁকিপূর্ণ কারণগুলি সম্পর্কে আপনার কি কোনও জ্ঞান আছে?)

| - Yes (হ্যাঁ) | - No (না) |
| --- | --- |

10. Do you know about any one of the following risk factors of Cervical Cancer? (সার্ভিকাল ক্যান্সারের নিম্নলিখিত যে কোনও একটি ঝুঁকির কারণ সম্পর্কে আপনি কি জানেন?)

| Weakened immune system (রোগ প্রতিরোধ ব্যবস্থা দুর্বল হয়ে পড়েছে) |  Yes (হ্যাঁ) |  No (না) |
| --- | --- | --- |
| Infection with HPV (Human Papilloma Virus) (এইচপিভি সংক্রমণ (মানব  প্যাপিলোমা ভাইরাস)) |  Yes (হ্যাঁ) |  No (না) |
| Long term use of contraceptive pill (গর্ভনিরোধক বড়ির দীর্ঘমেয়াদী ব্যবহার) |  Yes (হ্যাঁ) |  No (না) |
| Not going for regular  Pap test (নিয়মিত প্যাপ পরীক্ষা না করা) |  Yes (হ্যাঁ) |  No (না) |
| Having many children (অনেক বাচ্চা নেয়া) |  Yes (হ্যাঁ) |  No (না) |
| Having many sexual partners (অনেক যৌন সঙ্গী থাকা) |  Yes (হ্যাঁ) |  No (না) |
| HIV infection (এইচআইভি সংক্রমণ) |  Yes (হ্যাঁ) |  No (না) |
| Starting to have sex at a young age (before age 17) (অল্প বয়সে সেক্স শুরু করা (১৭ বছর বয়সের আগে)) |  Yes (হ্যাঁ) |  No (না) |

***Knowledge about the Symptoms of Cervical Cancer*** (***জরায়ু ক্যান্সারের লক্ষণ সম্পর্কে জ্ঞান)***

11. Do you have any idea about the symptoms of Cervical Cancer? (সার্ভিকাল ক্যান্সারের লক্ষণগুলি সম্পর্কে আপনার কোনও ধারণা আছে?)

| - Yes (হ্যাঁ) | - No (না) |
| --- | --- |

12. Do you have any knowledge about any one of the following symptoms of Cervical Cancer? (সার্ভিকাল ক্যান্সারের নিম্নলিখিত কোনও লক্ষণ সম্পর্কে আপনার কি কোনও জ্ঞান আছে?)

| Bleeding in between period (মাসিক ছাড়াই রক্তপাত) |  Yes (হ্যাঁ) |  No (না) |
| --- | --- | --- |
| Blood in the stool or urine (মল বা প্রস্রাবের সাথে রক্ত) |  Yes (হ্যাঁ) |  No (না) |
| Menstrual periods that are  longer than as usual (ঋতুস্রাব  স্বাভাবিকের চেয়ে দীর্ঘতর হওয়া) |  Yes (হ্যাঁ) |  No (না) |
| Persistent lower back pain & diarrhea (ক্রমাগত পিঠে ব্যাথা এবং ডায়রিয়া হওয়া) |  Yes (হ্যাঁ) |  No (না) |
| Unexplained weight loss (অব্যক্ত ওজন হ্রাস হওয়া) |  Yes (হ্যাঁ) |  No (না) |
| Vaginal bleeding after menopause (মেনোপজের পরেও যোনিপথে রক্তক্ষরণ) |  Yes (হ্যাঁ) |  No (না) |
| Persistent vaginal discharge that smells unpleasant (ক্রমাগত যোনি স্রাব যা থেকে অপ্রিয় গন্ধ করে ) |  Yes (হ্যাঁ) |  No (না) |
| Swollen legs, bone fractures (ফুলে যাওয়া পা, হাড়ভাঙা) |  Yes (হ্যাঁ) |  No (না) |

***Knowledge about Cervical Cancer Treatment*** (***জরায়ু ক্যান্সার*** ***চিকিৎসা সম্পর্কে জ্ঞান)***

13. Do you have any idea about the treatment of Cervical Cancer? (সার্ভিকাল ক্যান্সারের চিকিৎসা সম্পর্কে আপনার কোনও ধারণা আছে?)

| - Yes (হ্যাঁ) | - No (না) |
| --- | --- |

14. Do you know about any one of the following treatments of Cervical Cancer? (সার্ভিকাল ক্যান্সারের নিম্নলিখিত যে কোনও একটি চিকিৎসা সম্পর্কে আপনি কি জানেন?)

| Radiotherapy (রেডিওথেরাপি) |  Yes (হ্যাঁ) |  No (না) |
| --- | --- | --- |
| Chemotherapy (কেমোথেরাপি) |  Yes (হ্যাঁ) |  No (না) |
| Surgery (সার্জারি) |  Yes (হ্যাঁ) |  No (না) |
| Radical trachelectomy (র‌্যাডিকাল ট্র্যাচেকল্টোমি) |  Yes (হ্যাঁ) |  No (না) |
| Targeted therapy (লক্ষ্যযুক্ত থেরাপি) |  Yes (হ্যাঁ) |  No (না) |
| Radical hysterectomy (র‌্যাডিকাল হিস্টেরেক্টমি) |  Yes (হ্যাঁ) |  No (না) |
| Pelvic exenteration (শ্রোণী এক্সেনটিরেশন) |  Yes (হ্যাঁ) |  No (না) |
| Bilateral salpingo‐oophorectomy (দ্বিপাক্ষিক সালপিংও ‐ ওফোরেক্টমি) |  Yes (হ্যাঁ) |  No (না) |

***Knowledge about the Screening methods Cervical Cancer*** (***সার্ভিকাল ক্যান্সার সম্পর্কিত স্ক্রিনিং পদ্ধতি সম্পর্কে জ্ঞান)***

15. Do you ever heard about the screening methods of Cervical Cancer? (আপনি কি সার্ভিকাল ক্যান্সারের স্ক্রিনিং পদ্ধতি সম্পর্কে শুনেছেন?)

| o Yes (হ্যাঁ) | o No (না) |
| --- | --- |

16. Do you know about any one of the following screening methods of Cervical Cancer? (সার্ভিকাল ক্যান্সারের নিম্নলিখিত যে কোনও একটি স্ক্রিনিং পদ্ধতি সম্পর্কে আপনি কি জানেন?)

| Colposcopy (visual inspection of the cervix using a dilute acetic acid) (কলপোস্কোপি (পাতলা অ্যাসিটিক অ্যাসিড ব্যবহার করে জরায়ুর দৃষ্টিভঙ্গি পরিদর্শন)) |  Yes (হ্যাঁ) |  No (না) |
| --- | --- | --- |
| Ultrasound (আল্ট্রাসাউন্ড) |  Yes (হ্যাঁ) |  No (না) |
| Pelvic Exam (শ্রোণী পরীক্ষা) |  Yes (হ্যাঁ) |  No (না) |
| Pap Test (প্যাপ পরীক্ষা) |  Yes (হ্যাঁ) |  No (না) |
| HPV (Human Papillomavirus) Test (এইচপিভি (হিউম্যান প্যাপিলোমা ভাইরাস) পরীক্ষা) |  Yes (হ্যাঁ) |  No (না) |
| Endocervical curettage (A procedure to collect cells/tissue from the cervical canal) (এন্ডোসার্ভিকাল কুর্যারেজ (একটি পদ্ধতি জরায়ু খাল থেকে কোষ / টিস্যু সংগ্রহ করুন)) |  Yes (হ্যাঁ) |  No (না) |
| Cone Biopsy (removal of a large, cone shaped sample of cervical tissue) (শঙ্কু বায়োপসি (একটি বৃহৎ শঙ্কু অপসারণ)জরায়ুর টিস্যুর আকারের নমুনা)) |  Yes (হ্যাঁ) |  No (না) |
| CT scan (সিটি স্ক্যান) |  Yes (হ্যাঁ) |  No (না) |
| MRI (এমআরআই) |  Yes (হ্যাঁ) |  No (না) |
